# Elucidating the endophytic bacterial and fungal community composition and diversity in the tree fern *Alsophila spinulosa* through meta-amplicon sequencing

**DOI:** 10.3389/fmicb.2024.1445315

**Published:** 2024-08-29

**Authors:** Xiaohong Chen, Mengke Dou, Yuanhui Li, Jialan Su, Anjiu Zhao, Xiong Huang

**Affiliations:** ^1^College of Forestry, Sichuan Agricultural University, Chengdu, China; ^2^Ganzi Prefecture Forestry Research Institute, Kangding, China

**Keywords:** *Alsophila spinulosa*, endophytic bacteria, endophytic fungi, high-throughput sequencing, co-occurrence network

## Abstract

Plant tissues harbor abundant endophytes, which are crucial for plant growth. Endophytes present in *Alsophila spinulosa*, which is enriched with medicinal components, have not been isolated and characterized yet. Here we employed meta-amplicon sequencing to identify endophytic species and examined their diversity in the leaves, petioles, roots and stems of *A. spinulosa*. Our findings revealed 1,247 operational taxonomic units (OTUs) for endophytic bacteria across 210 species and 476 OTUs for endophytic fungi across 222 species. Alpha diversity analysis showed the highest endophytic bacterial diversity in *A. spinulosa* roots, whereas fungal diversity was similar across the leaf, petiole and root tissues. Fungal diversity in the leaves and petioles was markedly higher than that in the stems. Furthermore, beta diversity analysis revealed similarities in the endophytic bacterial and fungal compositions between the leaves and petioles, whereas the compositions in roots and stems considerably differed from those in the leaves and petioles. At the genus level, the predominant endophytic bacteria were *Methylobacterium-Methylorubrum* and *Pseudomonas*, whereas the predominant endophytic fungi were *Cutaneotrichosporon* and *Pseudofabraea*. Linear discriminant analysis effect size revealed characteristic endophytic bacterial genera specific to each tissue type and characteristic endophytic fungal genera specifically in the leaves, petioles and roots. The co-occurrence network analysis indicated that the complexity of endophyte networks was the highest in the leaves and the lowest in the stems of *A. spinulosa*. Overall, this study elucidates the distribution patterns of endophytes in *A. spinulosa* across various tissues, offering valuable microbial resources for the development of natural products for medicinal application.

## Introduction

Endophytes are microorganisms that inhabit the cells and interstitial spaces of various plant organs, including roots, stems, leaves, flowers, fruits, and seeds (Yan et al., 2022). These microorganisms can be present in plants at any stage of their lifecycle without causing visible disease symptoms (Liang et al., 2023). Endophytes are present either as symbiotic microbes that engage in beneficial or neutral interactions or as latent pathogens that reside in the host without causing immediate harm (Omomowo and Babalola, 2019). Fungi and bacteria are the predominant microbial types functioning as endophytes (Asad et al., 2023). Endophytes have synergistically evolved with their host plants, forming dynamic and balanced symbiotic relationships, which are beneficial to plant growth, resilience, and resistance to pests and diseases; in particular, endophytes promote nitrogen fixation, secrete growth hormones, and produce active metabolites to support plant growth (Kushwaha et al., 2020). Endophytes can be isolated from surface-sterilized plant tissues or directly detected within the tissues by using microbial DNA (macrogenomic) extraction techniques. Recently, numerous endophytes have been isolated and characterized from the roots, stems, and leaves of various species, including citrus trees, tea trees, grapevines, and *Taxus*. Endophytic species are the most abundant in the roots of forest trees, followed by stems and leaves (Abdalla and Matasyoh, 2014; Lin et al., 2022).

Medicinal plants are used to prevent and treat diseases (Aye et al., 2019). Either the whole plants or their specific parts serve as medicinal agents or raw materials in the pharmaceutical industry, providing diverse medicinal and economic benefits (Tang et al., 2023). Despite rapid advancements in modern medicine, including the methods for extracting metabolites from plants and other natural sources, synthetic chemistry, combinatorial chemistry, and molecular modeling for drug discovery (Ley and Baxendale, 2002; Mario Geysen et al., 2003; Lombardino and Lowe, 2004), medicinal plants, with their distinct advantages, continue to play a valuable role in disease treatment. They are not only culturally accepted and highly compatible with the human body but also more effective than synthetic compounds. Thus, natural products extracted from medicinal plants are still used in the synthesis of many clinical drugs (Tang et al., 2023). Endophytes have been isolated from various medicinal plants (Zhou et al., 2022; Zotchev, 2024). Many endophytes can secrete phytohormones, growth factors, and other compounds that support the growth and development of plants (Rani et al., 2022). In addition, endophytes regulate the accumulation and production of active ingredients in medicinal plants (Tshikhudo et al., 2023; Hashem et al., 2023). Endophytes are a source of many bioactive metabolites, including alkaloids, saponins, quinones, flavonoids, and terpenoids. These compounds are highly bioactive and have attracted substantial attention in natural drug research (Gouda et al., 2016). Thus, identifying the endophytes present in medicinal plants and understanding their effect on the physiology and biochemistry of these plants are essential for discovering new bioactive compounds.

*Alsophila spinulosa*, a member of the genus *Alsophila* in the Cyatheaceae family, is the only woody fern and often referred to as “the king of ferns” (Ma et al., 2020). It is classified as a second-class protected species under the newly issued List of Wild Plants of National Key Protection. *A. spinulosa* has become a hotspot of research in diverse fields, such as paleontology, paleobotany, paleogeology, paleoclimate, and paleoenvironmental changes (Loiseau et al., 2020). Moreover, some metabolites found in *A. spinulosa* exhibit antitumor and antibacterial properties (Longtine and Tejedor, 2017). The stems of *A. spinulosa*, containing flavonoids, phenols, amino acids, and alkaloids, are used in traditional Chinese medicine (Huang et al., 2022). Traditionally, *A. spinulosa* has been valued for its medicinal properties that include removing dampness, strengthening tendons and bones, activating blood circulation, and clearing heat and toxins (Chiang et al., 1994; Chaparro-Hernández et al., 2022), earning it the nickname “Dragon’s Bone Wind.” Existing studies on *A. spinulosa* have mainly focused on its population distribution, physiological ecology, medicinal components, and phylogenetic relationships (Xiong et al., 2023; Yi et al., 2023). However, no study has yet examined the distribution patterns of its endophytic composition, which hinders our comprehensive understanding of interactions between *A. spinulosa* and its endophytes as well as efforts to conserve the species and enhance its quality.

In the present study, we isolated and identified endophytes from the leaves, petioles, roots, and stems of *A. spinulosa*. Using meta-amplicon sequencing, we systematically cataloged endophyte species and their diversity across various plant parts and determined the distribution characteristics of the endophyte population. The findings of this study provide a scientific basis for further exploration and use of endophytic resources in *A. spinulosa*.

## Materials and methods

### Sample collection

For microbiome research, *A. spinulosa* tissues were collected from the National Germplasm Resource Center (29.9°N, 103.14°E), located in Hongya County, Sichuan Province, China. Mature tissues of the roots (R), stems (S), leaves (L), and petioles (P) collected from three individual trees were used as biological replicates. The collected samples were first rinsed with running water and then subjected to surface sterilization. The samples were sterilized first with 75% ethanol for 1 min and then with 5% sodium hypochlorite for 5 min. Subsequently, the samples were rinsed twice with sterilized deionized water and dried using sterilized filter paper. The final rinse water was used to verify the sterility of tissue surfaces through both polymerase chain reaction (PCR) amplification of 16S rRNA and internal transcribed spacer (ITS) genes and the plate cultivation method (Seghers et al., 2004). The verified tissue samples were placed in 50-mL sterilized centrifuge tubes, immediately frozen in liquid nitrogen, and stored at −80°C for 1 week.

### DNA extraction, library construction, and sequencing

We extracted total genomic DNA from *A. spinulosa* samples by using a HiPure Soil DNA Kit (Magen, Guangzhou, China) in accordance with the manufacturer’s instructions. DNA concentration and purity were assessed using a NanoDrop NC2000 spectrophotometer (Thermo Fisher Scientific, Waltham, MA, United States) and agarose gel electrophoresis, respectively. The extracted DNA was diluted to a concentration of 50 ng/μL with nuclease-free water. PCR amplification was performed using specific primer pairs for 16S rRNA (515F: GTGYCAGCMGCCGCGGTAA, 806R: GGACTACNVGGGTWTCTAAT) and ITS regions (ITS1F: CTTGGTCATTTAGAGGAAGTAA, ITS2: GCTGCGTTCTTCATC GATGC). The PCR reagents were obtained from New England Biolabs (USA). The initial PCR mixture consisted of 10 μL of Q5 reaction buffer (5×), 10 μL of Q5 High GC Enhancer (5×), 1.5 μL of 2.5 mM dNTPs, 1.5 μL of 10 μM each of forward and reverse primers, 0.2 μL of Q5 high-fidelity DNA polymerase, 1 μL of template DNA, and 24.3 μL of ddH_2_O. The PCR protocol involved an initial denaturation at 95°C for 5 min, followed by 30 cycles of denaturation at 95°C for 1 min, annealing at 60°C for 1 min, and extension at 72°C for 1 min, with a final extension at 72°C for 7 min. The PCR products were checked by gel electrophoresis (Supplementary Figures S1, S2) and purified from a 2% agarose gel using AMPure XP Beads (Beckman Coulter, Brea, CA, United States). DNA concentrations of the purified products were measured using a Qubit 3.0 Fluorometer (Thermo Fisher Scientific, Waltham, MA, United States), and these products were used as templates for the second round of PCR by using the same primers. The second-round PCR mixture comprised 5 μL of Q5 reaction buffer (5×), 1.5 μL of Q5 high GC enhancer (5×), 1.5 μL of 2.5 mM dNTPs, 1 μL of 10 mM each of index and universal PCR primers, 1 μL of Q5 high-fidelity DNA polymerase, and 1 μL of template DNA; 8 μL of ddH_2_O was added to make up the reaction volume to 20 μL. PCR protocol for the second round was as follows: initial denaturation at 95°C for 5 min, 12 cycles of denaturation at 95°C for 1 min, annealing at 60°C for 1 min, extension at 72°C for 1 min, and a final extension at 72°C for 7 min. The amplicons were purified, quantified using an ABI StepOnePlus Real-Time PCR System (Life Technologies, Foster City, United States), pooled in equal amounts, and subjected to high-throughput amplicon sequencing on an Illumina Novaseq 6,000 platform (Illumina, San Diego, CA, United States) to generate paired-end reads of approximately 250 bp. Totally, we obtained 1,526,330 and 1,533,219 raw reads for 16S rRNA and ITS sequencing, respectively (Supplementary Tables S1, S2).

### Bioinformatics and statistical analysis

The obtained raw 16S rRNA and ITS gene sequences were processed using the Quantitative Insights into Microbial Ecology pipeline (QIIME; version 1.9.0) (Caporaso et al., 2010). Initially, the sequences that contained any ambiguous bases, had more than two mismatches to the primers, had one mismatch to the barcode, and were shorter than 200 bp or had an average quality score of <20 were discarded. After filtering out these sequences and removing chimeras, the operational taxonomic units (OTUs) were picked using USEARCH (version 7.0.1090) at a 97% sequence identity threshold. Singletons were filtered out, and taxonomic annotation for the representative 16S rRNA and ITS sequences was performed. This was based on the Greengenes database (version 13.5) and UNITE database (version 7.0) using the RDP Classifier’s Bayes algorithm, with a confidence threshold of 70% (Goyer et al., 2022). Alpha diversity, which measures the variety within a single sample, was calculated and visualized using these tools. Beta diversity, which indicates the similarity between microbial communities across different samples, was also calculated using QIIME (version 1.9.0). To determine significant differences in microbial taxa among different tissue types, we performed linear discriminant analysis (LDA) effect size (LEfSe) (Segata et al., 2011), with a cutoff for discriminative features set at a logarithmic LDA score of 3.5.

The number of unique and common endophytic bacterial/fungal OTUs across different tissues of *A. spinulosa* was compared through Venn analysis, which was conducted using the VennDiagram package (Chen and Boutros, 2011) in R (version 3.6.0) (R Core Team, 2019). This analysis provided a preliminary understanding of the species composition of each subgroup. Diversity indices, namely Chao1, ACE, Shannon, Simpson, Good’s coverage, and Pielou’s evenness, were calculated using QIIME (version 1.9.0). Alpha diversity indices of *A. spinulosa* endophytes were visualized through box line plotting in Origin (version 2022). To determine significant differences in these indices among different tissues, we used the package “best Normalize” (Peterson, 2021) in R (version 3.6.0) to transform the data normality, after passing the test of homogeneity of variances (Supplementary Tables S3, S4), we conducted LSD multiple comparisons in SPSS (version 27.0). We investigated the endophytic diversity across different tissues. A *p* < 0.05 indicated statistical significance. The community composition of different subgroups is displayed in a stacked bar plot, created using the ggplot2 package (Kahle and Wickham, 2013) in R (version 3.6.0). In addition, the hierarchical cluster analysis and principal coordinates analysis (PCoA) of Bray–Curtis distances were performed using the Vegan package (Huffman et al., 2002) in R (version 3.6.0). The results highlighting similarities and differences in community composition between the samples were plotted using the ggplot2 package (Kahle and Wickham, 2013) in R (version 3.6.0).

To simplify network complexity, we selected genera with a relative abundance of >1.0% in the entire dataset as well as in each tissue type of *A. spinulosa*. Accordingly, co-occurrence networks were constructed (Fan et al., 2019). The Pearson’s correlation coefficients and *p*-values were calculated using the corr.test function of the psych package (Revelle and Revelle, 2015) in R (version 3.6.0). A Pearson’s correlation coefficient between two genera was deemed statistically significant if it exceeded 0.6 (Zhu et al., 2019) and had a *p* < 0.05 (Zhang et al., 2024). The co-occurrence network was built using the “igraph” package (Zhu et al., 2019) in R (version 3.6.0). Network modularization was determine using the “cluster_fast_greedy” function in the igraph package (Zhu et al., 2019). The topological parameters of the network were exported and visualized using Cytoscape (version 3.10.1). In this network, each node represents a genus, and each edge indicates a strong and significant correlation between different genera.

## Results

### Structural distribution of endophytic bacterial and fungal communities in *A. spinulosa* tissues

After filtering and optimizing the 12 sequenced samples, we obtained 1,562,330 high-quality bacterial sequences and 1,427,157 high-quality fungal sequences. Clustering based on 97% sequence similarity revealed 1,247 bacterial OTUs across 35 phyla, 90 classes, 205 orders, 308 families, 512 genera, and 210 species. In addition, we identified 476 fungal OTUs spanning 11 phyla, 31 classes, 74 orders, 222 families, 247 genera, and 222 species.

**Figure 1:**
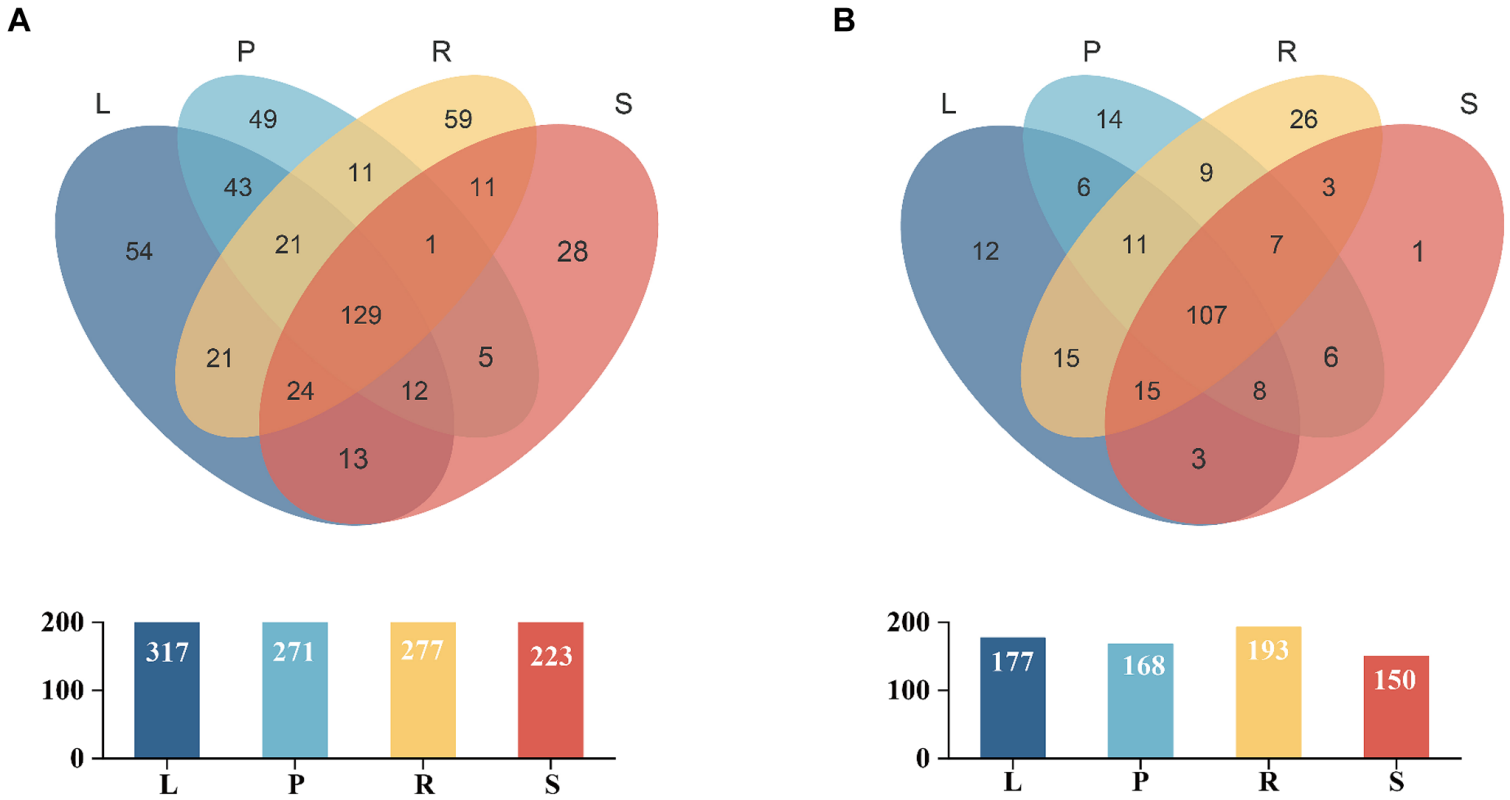
Venn diagram for the OTUs of bacterial **(A)** and fungal **(B)** populations in different parts of *A. spinulosa*. L, P, R, and S represent the leaves, petioles, roots, and stems of *A. spinulosa*, respectively. Different groups are represented by different colors, and numbers in the overlapping areas denote the number of species shared by the population.

The dilution curves for the bacterial and fungal sequences showed that the data plateaued at volumes of 8e+04 and 1e+05, respectively. This finding indicated that the sequencing data volume was adequate, ensuring the reliability and representativeness of the results for subsequent analyses (Supplementary Figure S3). Venn diagrams (Figure 1) present the distribution of endophytic bacterial and fungal OTUs across the samples. For endophytic bacteria, 481 OTUs were common across all four sample groups, with leaves containing the highest number of OTUs (*n* = 317), followed by roots (*n* = 277), petioles (*n* = 271), and stems (*n* = 223). Each plant part exhibited unique OTUs, with the root tissues exhibiting the highest number of unique species (*n* = 59), indicating a greater diversity of endophytic bacteria in roots (Figure 1A). Similarly, for endophytic fungi, 243 OTUs were common across the four groups, with roots again having the highest number of OTUs (*n* = 193), followed by leaves (*n* = 177), petioles (*n* = 168), and stems (*n* = 150). Each plant part had unique OTUs of fungi, with roots having the highest number of unique species (*n* = 26), suggesting a higher prevalence of endemic fungal species in roots (Figure 1B).

### Alpha diversity of endophytes in *A. spinulosa*

**Figure 2:**
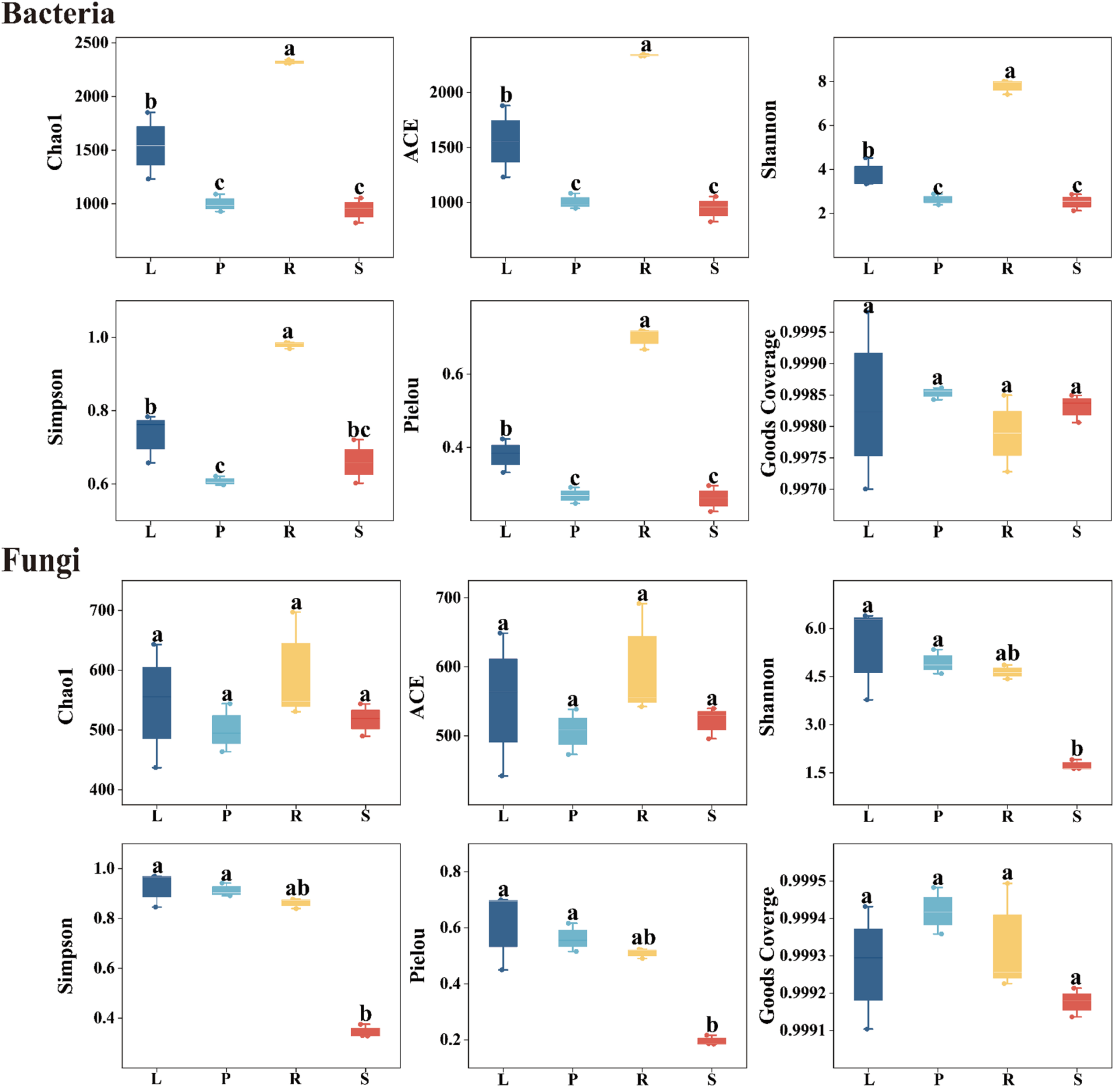
Alpha diversity of endophytic bacteria and fungi in different parts of *A. spinulosa*. L, P, R, and S represent the leaves, petioles, roots, and stems of *A. spinulosa*, respectively.

Alpha diversity is used to an indicator for species richness, evenness, and sequencing depth within a specific environment or ecosystem. Chao1 and Ace indices assess the richness of community microorganisms, with higher values indicating greater richness. Shannon and Simpson indices are used to evaluate the diversity of bacterial and fungal species, with higher values indicating highly diverse communities. Pielou index indicates the evenness or relative density of species, with higher values indicating greater evenness. Moreover, the Good’s coverage index assesses microbial coverage, with higher values suggesting a lower probability of missing new species in a sample. As shown in Figure 2, the sample coverage index for endophytic bacteria and fungi reached 1, suggesting that the sequencing depth covered nearly all species within the sample, which indicated that our data represent the actual community composition.

The Chao1 and Ace indices indicated that the richness of endophytic bacteria in *A. spinulosa* samples ranked in the order of roots > leaves > petioles > stems. This pattern suggests that the roots harbored the most endophytic bacteria, and stems had the least richness, with only slight differences in the bacterial abundance between petioles and stems. Similarly, the Shannon, Simpson, and Pielou indices for endophytic bacteria showed the sequence of roots > leaves > stems > petioles, indicating that the bacterial diversity and evenness were highest in the roots. By contrast, diversity and evenness in the leaves, petioles, and stems were similar but less pronounced. For endophytic fungi, the Chao1 and Ace indices did not differ significantly across the four tissue types of *A. spinulosa* (*p* > 0.05). However, the Shannon, Simpson, and Pielou indices revealed that the diversity and evenness of endophytic fungi in the leaves, petioles, and roots were similar but significantly higher than those in stems, indicating that stems have the lowest diversity and evenness of endophytic fungi.

### Beta diversity of endophytes in *A. spinulosa*

**Figure 3:**
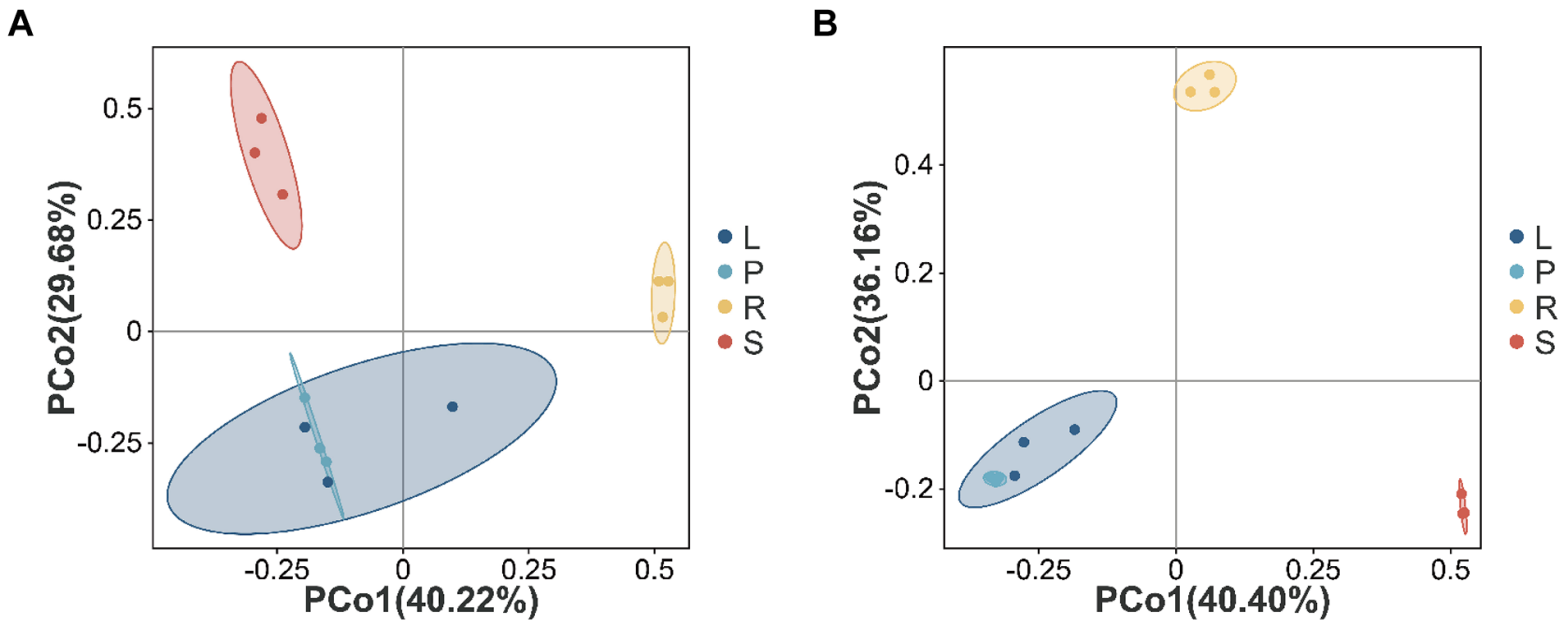
Beta diversity in different parts of *A. spinulosa*. The Bray–Curtis distance matrix for the principal coordinate analysis **(A,B)** of different endophytic bacterial and fungal communities in *A. spinulosa*. The distribution of endophytic bacterial **(A)** and fungal **(B)** communities. Each sample has three biological replicates. The values of the axes represent the percentage of variance that can be explained by each axis.

The PCoA method was used to analyze the beta diversity of *A. spinulosa* endophytes, which elucidated the similarities and differences in the community composition among different plant tissues. For endophytic bacterial communities (Figure 3A), PCo1 and PCo2 accounted for 40.22 and 29.68% of the variance, respectively. The bacterial communities from the roots and stems were primarily found in the first and second quadrants, distinguishing them from those in other plant parts. By contrast, the bacterial communities in the leaves and petioles clustered more closely, indicating similar structural compositions between these two parts. As depicted in Figure 3B, the endophytic fungal communities in the leaves and petioles also showed close clustering. In addition, distinct clustering of the samples based on the tissue type indicated the significant effect of tissue type on the endophyte community structure.

### Composition of endophytic bacterial and fungal communities

**Figure 4:**
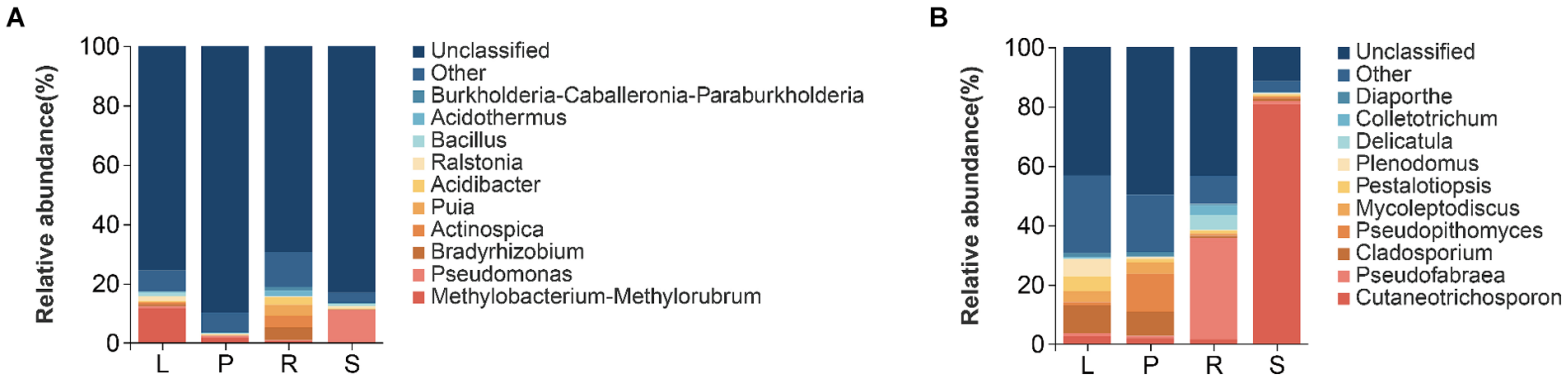
Histograms of relative richness at the genus level of bacteria **(A)** and fungi **(B)** in different parts of *A. spinulosa*.

The structure of the endophytic bacterial community in the four parts of *A. spinulosa* was analyzed at the genus level on the basis of OTU species nodes. The top 10 average richness in all samples were detailed, whereas the remaining species were uniformly categorized as “Other.” Tags that could not be classified at this level were grouped as “Unclassified” (Figure 4).

At the genus level, the relative richness of *Methylobacteria-methylorubrum* was the highest in the leaves (11.618%), followed by that of *Ralstonia* (1.582%) and *Bacillus* (1.315%). In petioles, *Methylobacteria-methylorubrum* was the most abundant (1.676%), followed by *Pseudomonas* (0.694%) and *Burkholderia-Caballeronia Paraburkholderia* (0.332%). Root communities predominantly contained *Bradyrhizobium* (4.257%), *Actinospica* (3.919%), and *Puia* (3.686%). However, *Pseudomonas* (10.830%) and *Bacillus* (0.892%) were abundant in the stems. The primary endophytic bacterial groups in the leaves, petioles, roots, and stems were *Methylobacteria-methylorubrum* and *Pseudomonas* (Figure 4A).

At the genus level of endophytic fungi, in leaves, the relative richness of *Cladosporium* was the highest (9.405%), followed by that of *Plenodomus* (5.847%), *Pestalotiopsis* (5.052%), and *Mycoleptodiscus* (3.682%). In petioles, *Pseudopithomyces* was the most abundant (12.785%), followed by *Cladosporium* (8.140%) and *Mycoleptodiscus* (3.720%). Additionally, *Pseudofabraea* (34.050%), *Delicatula* (5.012%), and *Colletotrichum* (3.243%) were the main root endophytic fungi, whereas *Cutaneotrichosporon* (80.789%) was the predominant stem endophytic fungus. The main endophytic fungal groups in the leaves, petioles, roots, and stems belonged to *Cutaneotrichosporon* and *Pseudofabraea* (Figure 4B).

The characteristic endophytic bacteria and fungi in the four parts of *A. spinulosa* were identified through LEfSe analysis. According to LDA with a threshold of ≥3.5, five characteristic genera of endophytic bacteria and eight characteristic genera of endophytic fungi were identified (Supplementary Figure S4). The characterized genera of endophytic bacteria included *Stenotrophomonas* from stems, *Pajaroellobacter* from roots, *Pelagibacteriu* from petioles, and *Methylobacterium_Methylorubrum* and *Sphingomonas* from leaves. Endophytic characteristic genera of fungi were *Pseudeurotium*, *Tulasnella*, and *Exophiala* from roots; *Periconia* and *Clonostachys* from petioles; and *Dichotomophthora*, *Cladosporium*, and *Coprinellus* from leaves. No endophytic fungi characterizing genera with LDA (log_10_) > 3.5 were noted in *A. spinulosa* stems.

### Network symbiotic structure of endophytic bacteria and fungi in *A. spinulosa*

The network structure of endophytic bacteria and fungi in *A. spinulosa* is characterized by modules, which are densely connected network areas where internal links outnumber external ones. After modularization, the endophytic bacterial community co-emergence network of the leaves and petioles was divided into 10 modules, whereas that of the roots and stems was divided into 8 and 7 modules, respectively. This finding suggested the presence of stronger connections among endophytic bacteria in the stems of *A. spinulosa* (Supplementary Figure S5). The endophytic fungal community co-emergence network of the petioles, roots, and stems had 11 modules each, whereas that of the leaves had eight modules, indicating stronger connections among endophytic fungi in the leaves of *A. spinulosa* (Supplementary Figure S6). Moreover, the modularity index of each symbiotic network exceeded 0.4, demonstrating that the endophytic bacterial and fungal networks in all parts of *A. spinulosa* exhibited a modular structure.

**Figure 5:**
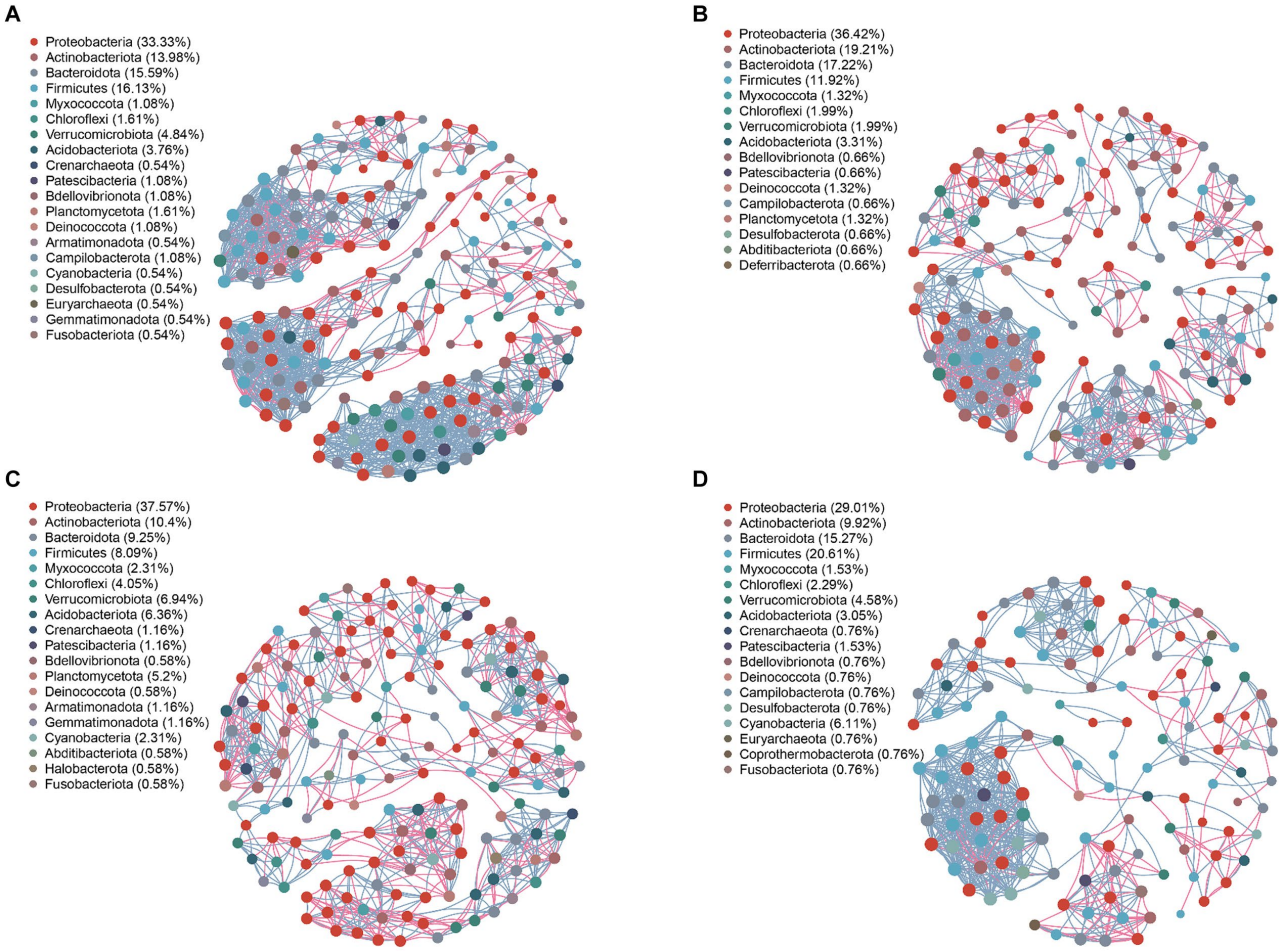
Symbiotic network characteristics of *A. spinulosa* endophytic bacteria in the leaves **(A)**, petioles **(B)**, roots **(C)**, and stems **(D)**. Genera with a relative abundance of >0.01 were selected, and the Pearson’s correlation coefficient between two genera was calculated. Based on correlation [Pearson’s coefficient >0.6 (correlation); *p* < 0.05 (significant)] relationships, a network map was drawn. The size of the node in the network is related to the connective degree of the point; the color represents the level of the phylum, and the red and sky blue lines represent the positive and negative correlations between the two nodes, respectively; the thicker the line, the stronger is the correlation. Different taxa are represented by different colors.

**Figure 6:**
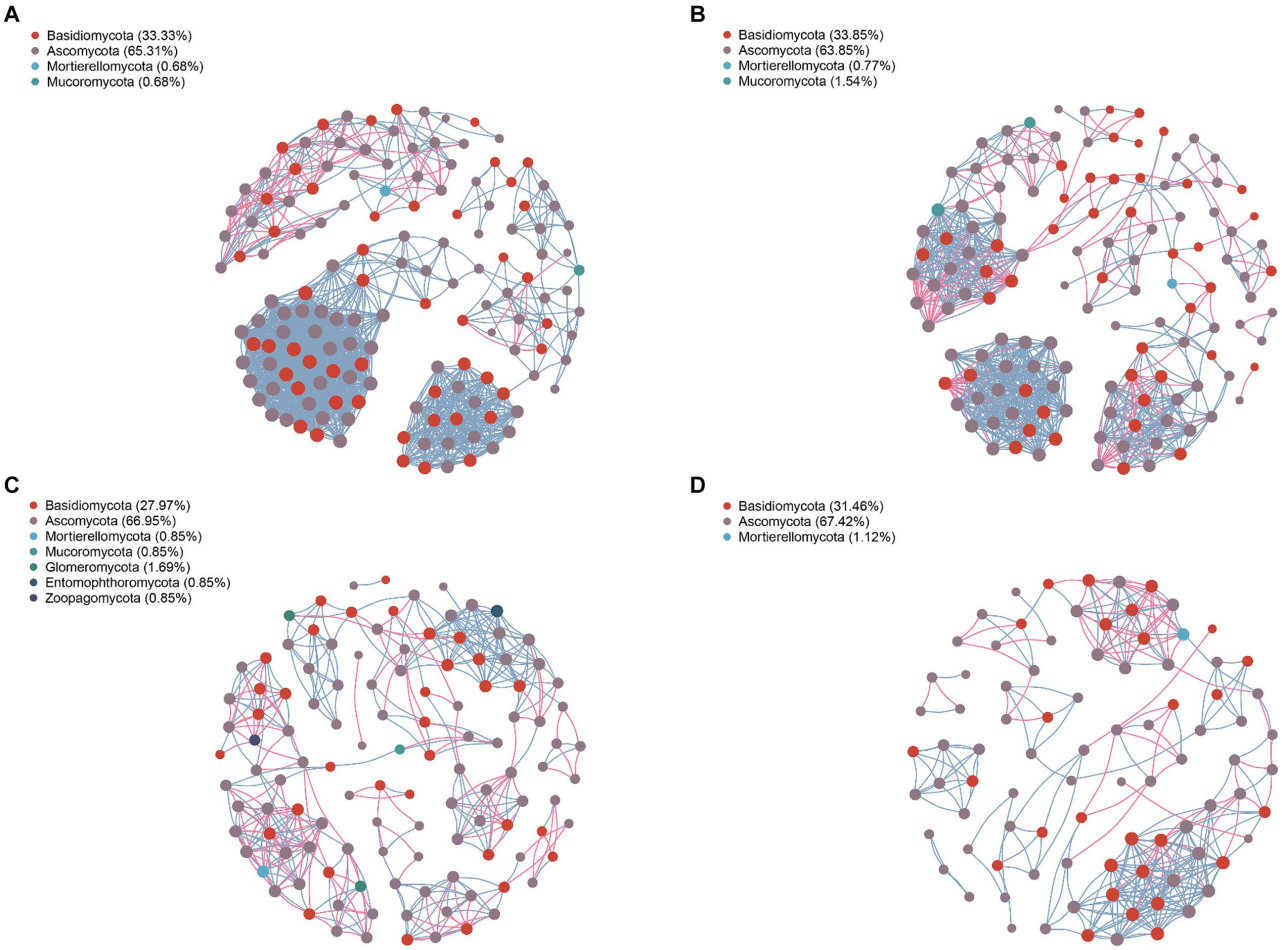
Symbiotic network characteristics of endophytic fungi in each part of *A. spinulosa*. **(A)** Leaves; **(B)** petioles; **(C)** roots; and **(D)** stems.

Analyzing the network nodes at the genus level according to phyla revealed the presence of 20, 16, 19, and 18 bacterial phyla in the community networks of the leaves, petioles, roots, and stems, respectively. Four phyla—Proteobacteria, Firmicutes, Bacteroidota, and Actinobacteriota—were particularly prevalent, accounting for 79.03, 84.77, 65.31, and 74.81% of all nodes in these communities, respectively (Figure 5). Similarly, the fungal community network of the leaves, petioles, roots, and stems was divided into 4, 16, 19, and 18 phyla, respectively. The phyla Basidiomycota and Ascomycota were predominantly distributed, accounting for 98.64, 97.7, 94.92, and 98.88% of all fungal nodes, respectively (Figure 6). These results indicate that the main endophytic bacteria and fungi were similarly distributed across the different parts of *A. spinulosa*.

To further understand the co-occurrence patterns of endophytic bacteria in each part of *A. spinulosa*, separate bacterial and fungal networks were constructed for the four sample types, and their topological properties were analyzed (Table 1). These properties elucidated the complex patterns of node correlations within the networks. The analysis of co-occurrence network characteristics revealed that the leaf bacterial community network exhibited the highest complexity, whereas the stem network exhibited the least complexity, indicating that the leaf network had the highest number of nodes and connections, whereas the stem network had the least number of nodes and connections. Specifically, the leaf endophytic bacterial community network comprised 186 nodes (genera) and 1,381 connections, whereas the fungal network in the same part included 147 nodes and 1,346 connections. By contrast, the stem bacterial network contained only 131 nodes and 665 connections, and its fungal counterpart had 89 nodes and 300 connections. Furthermore, the leaf community displayed predominantly positive symbiotic relationships, as indicated by the highest ratios of positive to negative correlation connections (P/N) in both bacterial (5.057) and fungal (13.956) networks. The root networks, both bacterial and fungal, exhibited longer paths, greater diameters, and better modularity, suggesting a stronger topological structure. Furthermore, the leaf endophytic bacterial network showed the highest average clustering coefficient (avgCC) and average degree (avgK), indicating superior network connectivity compared with those of the other parts. By contrast, the root bacterial network had lower avgCC and avgK, suggesting less connectivity in the root tissues compared with the other tissue types.

**Table 1:** Symbiotic network characteristics of endophytic bacterial and fungal communities in the leaf, petiole, root, and stem tissues of *A. spinulosa.*

Network properties	Endophytic bacteria				Endophytic fungi			
	Leaf	Petiole	Root	Stem	Leaf	Petiole	Root	Stem
Nodes	186	151	173	131	147	130	118	89
Edges	1,381	792	768	665	1,346	751	394	300
Modularity (MD)	0.691	0.715	0.803	0.634	0.548	0.702	0.782	0.673
P/N	5.057	2.650	1.469	4.783	13.956	3.579	1.680	2.797
Clustering coefficient (avgCC)	0.795	0.822	0.747	0.764	0.843	0.825	0.771	0.773
Average path length (APL)	3.112	2.598	17.117	3.522	2.530	3.064	9.680	4.477
Network diameter (ND)	12	9	50	14	10	13	28	16
Average degree (avgK)	14.849	10.490	8.879	10.153	18.313	11.554	6.678	6.742

## Discussion

In this study, we used 16S rRNA and ITS high-throughput sequencing to perform a differential and comparative analysis of the composition and diversity of endophytes communities in the leaves, petioles, roots, and stems of *A. spinulosa*. This methodology eliminates the need to isolate microorganisms from purified cultures, offering unique advantages in characterizing microbial communities. This method enables us to obtain a more comprehensive understanding of endophytes by allowing direct extraction of total DNA from the sample environment and its sequencing, thereby circumventing the limitations of traditional culture methods. This approach is particularly suitable for studying various samples, such as plant microorganisms (Glenn et al., 2019; Quijada et al., 2020). In our study, we identified 481 shared OTUs among the four sample groups at the genus level of endophytic bacterial genera in *A. spinulosa*, with leaves containing the highest number of OTUs. This finding may be attributed to microenvironmental differences across plant tissues, which affect the survival of endophytic bacteria (Lin et al., 2022). Specifically, the leaf microenvironment in *A. spinulosa* appeared to be more conducive to the survival of diverse endophytic bacteria, leading to a higher number of endophytic bacterial OTUs in leaves than in the other tissues.

Our alpha diversity analysis revealed that the diversity of endophytic bacteria in *A. spinulosa* varied across different tissues. Specifically, the roots of *A. spinulosa* displayed higher endophytic bacterial diversity than its leaf blades, petioles, and stems; however, the stems exhibited the lowest diversity of endophytic fungi. Furthermore, beta diversity analyses indicated that the endophytic bacterial communities in the roots and stems were distinct from those in the other plant parts. By contrast, endophytic bacterial communities in leaf blades and petioles were closely clustered. Petioles and leaf blades constitute the leaf tissues. The petiole serves as the conduit for water and nutrient transfer across the leaf blades and stems, in addition to providing support to the leaf blades for optimal sunlight exposure. Thus, the petioles and leaf blades are interconnected, leading to similarities in the structure of endophyte communities. This observation aligns with the conclusions drawn by Wei et al. (2018) and Ferreira-Silva et al. (2021), highlighting the variation in the distribution of endophyte communities across different tissues. The genetic characteristics of *A. spinulosa* might affect the diversity and structure of endophyte communities. Factors such as genetic traits and growth characteristics can lead to differences in the physical structure, chemical composition, and nutrient content of various tissues, thereby affecting the diversity and structure of endophyte communities in plants.

The structure of endophyte communities in plants is dynamic and affected by various abiotic and biotic factors, including soil conditions, biogeography, plant species, microbe–microbe interactions, and plant–microbe interactions, operating at both local and larger scales (García-Guzmán and Heil, 2014). During evolution, endophytes of *A. spinulosa* have developed a mutually beneficial symbiotic relationship with their host plants. The host plant provides photosynthesized products and minerals to endophytes, which, in turn, support the host plant’s growth, development, and phylogenetic evolution. Our study revealed high diversity of the endophytic bacteria, belonging to 35 phyla, 90 classes, 205 orders, 308 families, 512 genera, and 210 species, in *A. spinulosa*. The identified endophytic fungi were found to belong to 11 phyla, 31 classes, 74 orders, 222 families, 247 genera, and 222 species. These findings provide insights into the composition of endophytic bacterial and fungal communities in *A. spinulosa*.

At the genus level, *A. spinulosa* harbored endophytic bacteria with high abundance, including *Methylobacterium-Methylorubrum*, *Pseudomonas*, and *Bradyrhizobium*. Previous studies have reported the unique one-carbon metabolic pathway of *Methylobacterium*, which plays a crucial role in the biometabolic pathways and evolutionary diversity (Chistoserdova et al., 1998). *Pseudomonas* plays a crucial role in biocontrol (Schlatter et al., 2017) by protecting plants from ion-induced oxidative stress through enhancing nutrient availability, reducing ion uptake, and triggering plant antioxidant responses (Islam et al., 2014). In our study, the genus *Burkholderia-Caballeronia-Paraburkholderia* was detected in the petioles of *A. spinulosa*, whereas numerous slow-growing rhizobacteria were found in its roots. These bacteria alleviate nutrient stress in the host plant by performing nitrogen fixation and secreting growth hormones, particularly under relatively infertile conditions, thereby enhancing plant growth (Hara et al., 2019; Dudeja et al., 2021). Moreover, certain endophytic bacterial populations, such as *Pseudomonas* and *Bacillus*, contribute to the host’s resistance against pathogens, drought, salt, and other biotic and abiotic stresses (Ali et al., 2014; Naveed et al., 2014). The enrichment of these endophytic bacterial flora substantially promotes plant health, growth, and development. However, the mechanisms underlying their effects on the growth of *A. spinulosa* remain to be fully elucidated.

Most endophytic fungi belonging to the genera *Cutaneotrichosporon*, *Pseudofabraea*, and *Cladosporium* were distributed across all tissues—leaves, petioles, roots, and stems—of the *A. spinulosa* samples. *Cladosporium* species/strains produce secondary metabolites, which serve as a rich source of bioactive compounds, and these compounds are widely used as antimicrobials, anthelmintics, and anticancer agents (Salvatore et al., 2021). Moreover, *Cladosporium* can produce indole-3-acetic acid and/or sterols, which play a role in enhancing plant growth. Thus, further genome analysis of *Cladosporium* species can lead to the discovery of new metabolites and metabolic pathways associated strongly with plant growth (Yang et al., 2023). Endophytic fungi offer numerous benefits for plant growth, including nitrogen fixation, phosphorus solubilization, phytohormone production, root morphology alteration, osmotic regulation, increased iron phosphate carrier production, enhanced solubilizing activity, and stomatal regulation. However, limited studies on the genera *Cutaneotrichosporon* and *Pseudofabraea* have hindered our understanding of whether an intrinsic link exists between these endophytic fungal taxa and the healthy growth of *A. spinulosa* as well as their metabolite activities.

Network analysis offers a unique and comprehensive perspective on microbial interactions by allowing clear visualization of the various forms of interactions between different taxa within a microbial community (Barberán et al., 2012; Ziegler et al., 2018; Banerjee et al., 2019). Modularity index values exceeding 0.4 indicate a typical modular structure (Newman, 2006). In our study, all modularity index values were higher than 0.4, indicating a robust modular structure in the networks constructed by endophytic flora at each site. A robust modular structure not only helps the endophytes of *A. spinulosa* in resisting environmental changes but also enhances the stability of the interaction network (Krause et al., 2003). Ecological networks are typically characterized by diverse biological interactions, where species display complex positive and negative associations (Chow et al., 2014). Positive correlations indicate cooperative relationships among biological populations, whereas negative correlations indicate competitive or predatory relationships. Our findings revealed that the nodes and connections of endophytic bacterial and fungal networks were most abundant in the leaves. Moreover, the symbiotic network of leaf endophyte communities displayed the highest number of positive and negative correlations, suggesting that the endophyte network in *A. spinulosa* leaves were most complex and had positive symbiotic relationships. Finally, our results align with those of previous studies, which have indicated that endophytic bacteria in most plants belong mainly to the phyla Proteobacteria, Firmicutes, Bacteroidota, and Actinobacteriota (Hardoim et al., 2015) and that the endophytic fungal community is dominated by Ascomycota and Basidiomycota (Marques et al., 2015).

## Conclusion

The abundance, distribution, and diversity of endophytic bacteria in *A. spinulosa* are likely affected by both genetic characteristics and tissue types. Our study revealed the highest diversity of endophytic bacteria in *A. spinulosa* roots, and the diversity of endophytic fungi was lowest in the stems of *A. spinulosa*. Furthermore, leaf tissues showed the highest complexity of the endophyte network, whereas stems exhibited the lowest complexity of the mycorrhizal network. Some endophytes exhibit clear tissue preferences, unlike the endophytic bacterium *Methylobacterium-Methylorubrum* and the endophytic fungus *Cladosporium*, among others, which can parasitize various tissues. A limitation of the present study is the lack of a detailed examination of the practical application effects of endophytes in *A. spinulosa*. Identification of endophyte species in *A. spinulosa* can not only enhance our understanding of its microbial community composition and abundance but also provide insights into the symbiotic relationship between *A. spinulosa* and microorganisms. This study lays the theoretical groundwork for future research into its biological roles, particularly the biocontrol and antiretroviral properties of *A. spinulosa*.

## Data Availability

The datasets presented in this study can be found in online repositories. The names of the repository/repositories and accession number(s) can be found in the article/Supplementary material.
