# Supplementary material for: Elucidating the endophytic bacterial and fungal community composition and diversity in the tree fern Alsophila spinulosa through meta-amplicon sequencing
Source: Front Microbiol. 2024 Aug 29;15:1445315. doi: 10.3389/fmicb.2024.1445315 (PMC11390551; doi:10.3389/fmicb.2024.1445315)
Supplement: Supplementary file 1 [file Data_Sheet_1.docx]

Supplementary Material

Table S1 16S rRNA sequencing of 4 tissues in *A. spinulosa*. L: leaf; P: petiole; R: root; S: stem.

| Sample Name | Raw Reads | Clean Reads | Raw Tags | Clean Tags | Chimera | Effective Tags | Effective Ratio (%) |
| --- | --- | --- | --- | --- | --- | --- | --- |
| L1 | 129547 | 129170 | 127246 | 126659 | 12427 | 114232 | 88.18 |
| L2 | 122664 | 121518 | 119055 | 118411 | 3540 | 114871 | 93.65 |
| L3 | 131100 | 130168 | 127808 | 127136 | 6683 | 120453 | 91.88 |
| P1 | 131500 | 131185 | 129097 | 128373 | 4477 | 123896 | 94.22 |
| P2 | 131604 | 131382 | 129125 | 128373 | 6093 | 122280 | 92.92 |
| P3 | 137478 | 137296 | 135004 | 134244 | 6126 | 128118 | 93.19 |
| R1 | 131027 | 130844 | 128807 | 128214 | 11901 | 116313 | 88.77 |
| R2 | 137431 | 136898 | 134706 | 133733 | 12560 | 121173 | 88.17 |
| R3 | 122289 | 122081 | 120226 | 119307 | 14027 | 105280 | 86.09 |
| S1 | 136075 | 135499 | 133165 | 132469 | 6958 | 125511 | 92.24 |
| S2 | 120746 | 119180 | 117069 | 116471 | 4447 | 112024 | 92.78 |
| S3 | 130869 | 130519 | 128285 | 127647 | 5358 | 122289 | 93.44 |

Table S2 ITS sequencing of 4 tissues in *A. spinulosa*. L: leaf; P: petiole; R: root; S: stem.

| Sample Name | Raw Reads | Clean Reads | Raw Tags | Clean Tags | Chimera | Effective Tags | Effective Ratio (%) |
| --- | --- | --- | --- | --- | --- | --- | --- |
| L1 | 132863 | 132573 | 122332 | 121136 | 396 | 120740 | 90.88 |
| L2 | 124577 | 124274 | 117113 | 116204 | 28 | 116176 | 93.26 |
| L3 | 121524 | 121246 | 112747 | 111603 | 130 | 111473 | 91.73 |
| P1 | 120983 | 120835 | 116500 | 115714 | 159 | 115555 | 95.51 |
| P2 | 129601 | 129401 | 123198 | 122281 | 230 | 122051 | 94.17 |
| P3 | 132558 | 132398 | 128405 | 127639 | 240 | 127399 | 96.11 |
| R1 | 124680 | 124374 | 113635 | 112473 | 584 | 111889 | 89.74 |
| R2 | 134960 | 134637 | 123653 | 122491 | 732 | 121759 | 90.22 |
| R3 | 129124 | 128814 | 118572 | 117628 | 764 | 116864 | 90.51 |
| S1 | 130292 | 129238 | 125092 | 123110 | 32 | 123078 | 94.46 |
| S2 | 123279 | 122465 | 119265 | 118363 | 61 | 118302 | 95.96 |
| S3 | 128778 | 128472 | 122710 | 121978 | 107 | 121871 | 94.64 |

| Table S3 Test of homogeneity of variances for endophytic bacterial diversity indices. | | | | | |
| --- | --- | --- | --- | --- | --- |
|  |  | Levene Statistic | df 1 | df 2 | Sig. |
| Shannon | Based on Mean | 2.342 | 3 | 8 | 0.149 |
|  | Based on Median | 0.203 | 3 | 8 | 0.892 |
|  | Based on Median and with adjusted df | 0.203 | 3 | 3.636 | 0.889 |
|  | Based on trimmed mean | 2.001 | 3 | 8 | 0.193 |
| Simpson | Based on Mean | 3.521 | 3 | 8 | 0.069 |
|  | Based on Median | 1.06 | 3 | 8 | 0.418 |
|  | Based on Median and with adjusted df | 1.06 | 3 | 3.575 | 0.467 |
|  | Based on trimmed mean | 3.291 | 3 | 8 | 0.079 |
| Chao1 | Based on Mean | 2.211 | 3 | 8 | 0.164 |
|  | Based on Median | 2.132 | 3 | 8 | 0.174 |
|  | Based on Median and with adjusted df | 2.132 | 3 | 3.02 | 0.274 |
|  | Based on trimmed mean | 2.208 | 3 | 8 | 0.165 |
| ACE | Based on Mean | 2.472 | 3 | 8 | 0.136 |
|  | Based on Median | 2.378 | 3 | 8 | 0.146 |
|  | Based on Median and with adjusted df | 2.378 | 3 | 2.863 | 0.254 |
|  | Based on trimmed mean | 2.469 | 3 | 8 | 0.136 |
| Goods Coverage | Based on Mean | 2.926 | 3 | 8 | 0.1 |
|  | Based on Median | 2.213 | 3 | 8 | 0.164 |
|  | Based on Median and with adjusted df | 2.213 | 3 | 2.876 | 0.271 |
|  | Based on trimmed mean | 2.884 | 3 | 8 | 0.103 |
| Pielou | Based on Mean | 0.508 | 3 | 8 | 0.688 |
|  | Based on Median | 0.29 | 3 | 8 | 0.831 |
|  | Based on Median and with adjusted df | 0.29 | 3 | 6.468 | 0.831 |
|  | Based on trimmed mean | 0.492 | 3 | 8 | 0.697 |

| Table S4 Test of homogeneity of variances for endophytic fungal diversity indices. | | | | | |
| --- | --- | --- | --- | --- | --- |
|  |  | Levene Statistic | df1 | df2 | Sig. |
| Shannon | Based on Mean | 3.016 | 3 | 8 | 0.094 |
|  | Based on Median | 0.742 | 3 | 8 | 0.556 |
|  | Based on Median and with adjusted df | 0.742 | 3 | 3.354 | 0.588 |
|  | Based on trimmed mean | 2.769 | 3 | 8 | 0.111 |
| Simpson | Based on Mean | 3.142 | 3 | 8 | 0.087 |
|  | Based on Median | 0.938 | 3 | 8 | 0.466 |
|  | Based on Median and with adjusted df | 0.938 | 3 | 3.187 | 0.516 |
|  | Based on trimmed mean | 2.929 | 3 | 8 | 0.1 |
| Chao1 | Based on Mean | 2.508 | 3 | 8 | 0.133 |
|  | Based on Median | 0.362 | 3 | 8 | 0.782 |
|  | Based on Median and with adjusted df | 0.362 | 3 | 3.356 | 0.786 |
|  | Based on trimmed mean | 2.235 | 3 | 8 | 0.162 |
| ACE | Based on Mean | 3.914 | 3 | 8 | 0.054 |
|  | Based on Median | 0.44 | 3 | 8 | 0.731 |
|  | Based on Median and with adjusted df | 0.44 | 3 | 3.203 | 0.74 |
|  | Based on trimmed mean | 3.381 | 3 | 8 | 0.075 |
| Goods Coverage | Based on Mean | 3.444 | 3 | 8 | 0.072 |
|  | Based on Median | 0.591 | 3 | 8 | 0.638 |
|  | Based on Median and with adjusted df | 0.591 | 3 | 4.495 | 0.65 |
|  | Based on trimmed mean | 3.069 | 3 | 8 | 0.091 |
| Pielou | Based on Mean | 3.016 | 3 | 8 | 0.094 |
|  | Based on Median | 0.742 | 3 | 8 | 0.556 |
|  | Based on Median and with adjusted df | 0.742 | 3 | 3.354 | 0.588 |
|  | Based on trimmed mean | 2.769 | 3 | 8 | 0.111 |


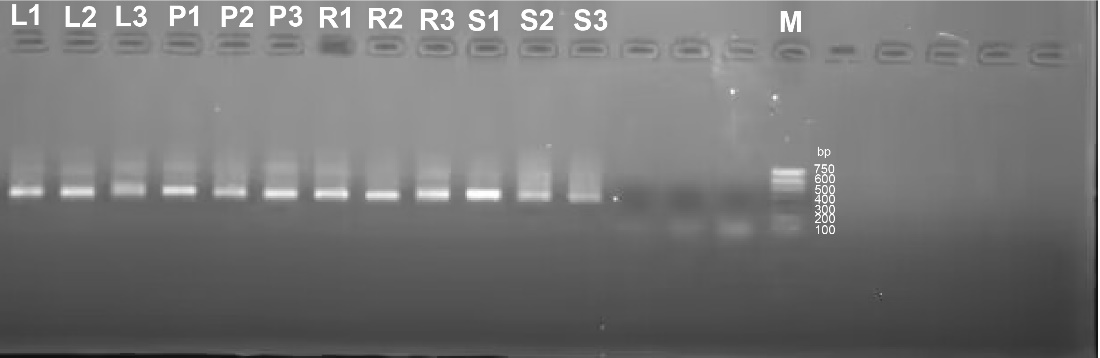


Figure S1 Gel electrophoresis of ITS PCR products. L: leaf; P: petiole; R: root; S: stem.


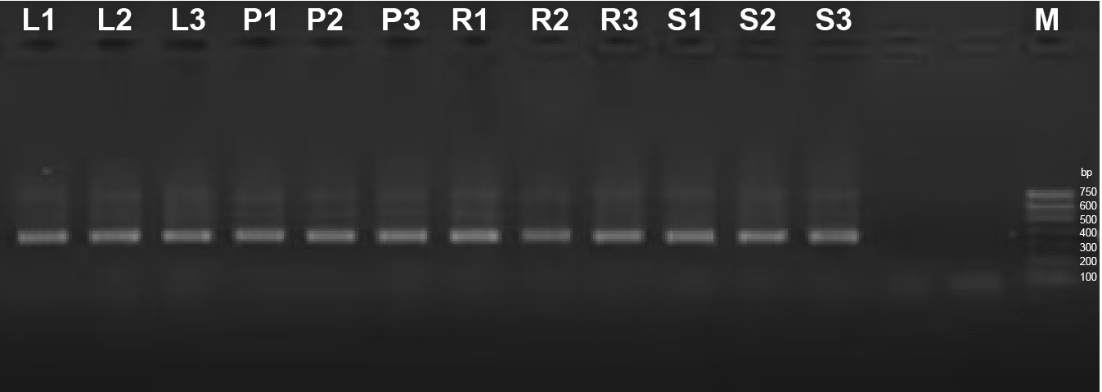


Figure S2 Gel electrophoresis of 16S rRNA PCR products. L: leaf; P: petiole; R: root; S: stem.


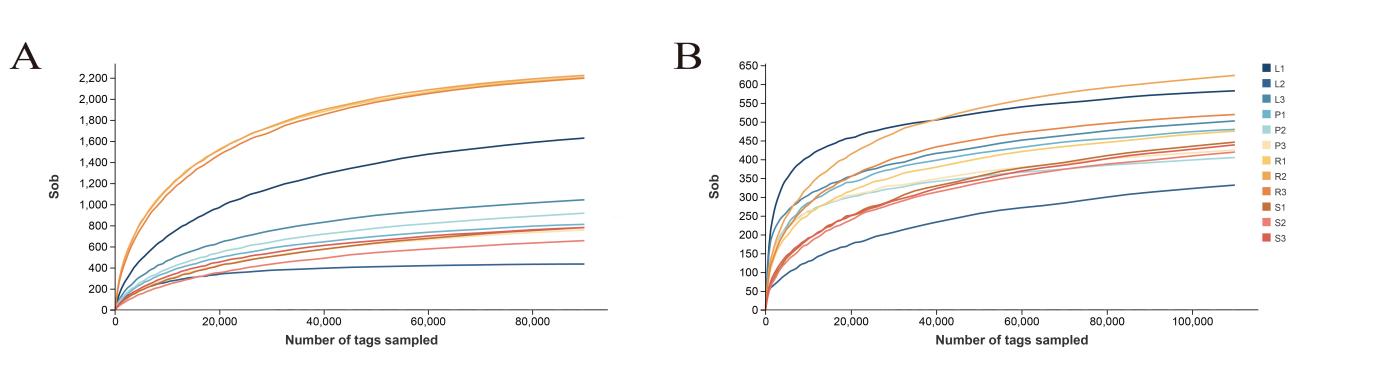


Figure S3 Rare curves of endophytic bacterial (A) and fungal (B) tags. L1-3、P1-3、R1-3、S1-3 represented three repeated samples of leaves, petioles, roots and stems, respectively.


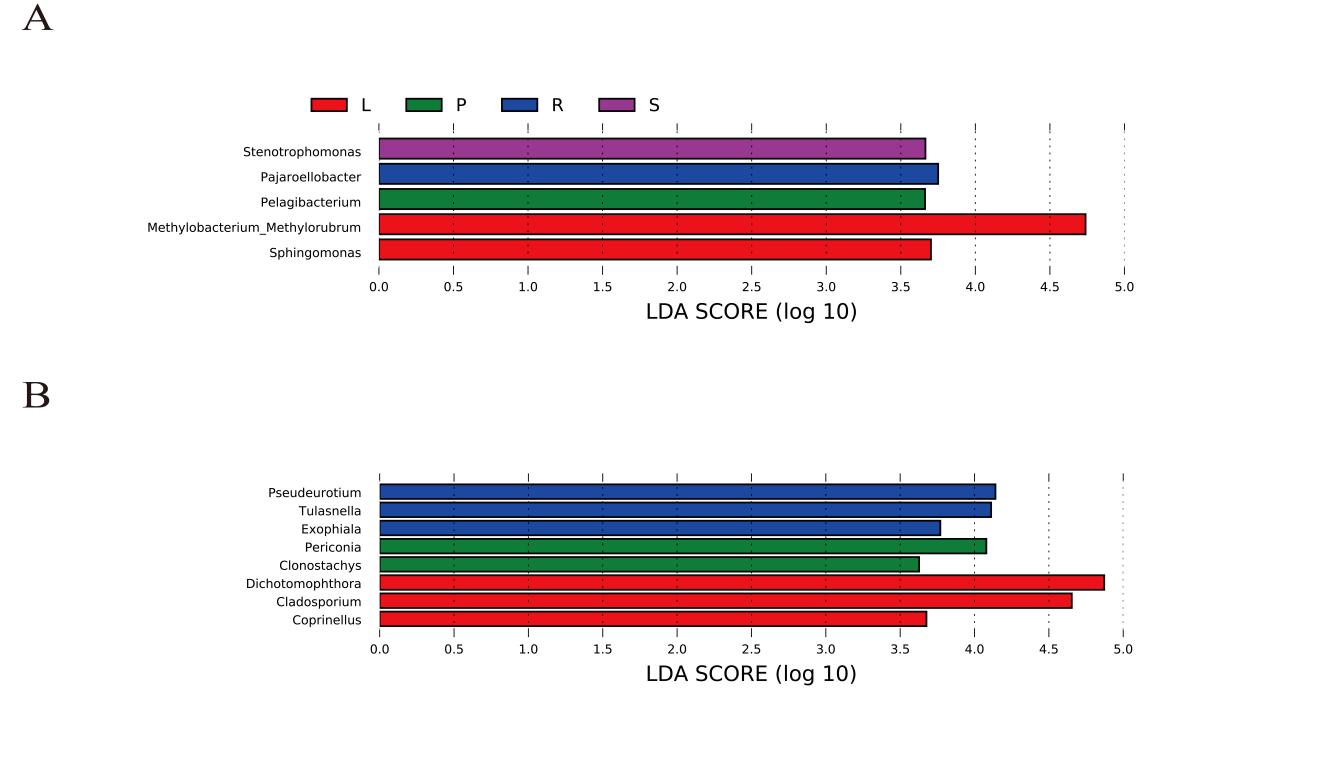


Figure S4 Characteristic genera identified by linear discriminant effect size (LEfSe) analysis that explains the differences of endophytic bacterial (A) and fungal (B) communities in different parts of *A. spinulosa*. The threshold on the logarithmic LDA score for discriminative features was set to 3.5 (*P*<0.05).


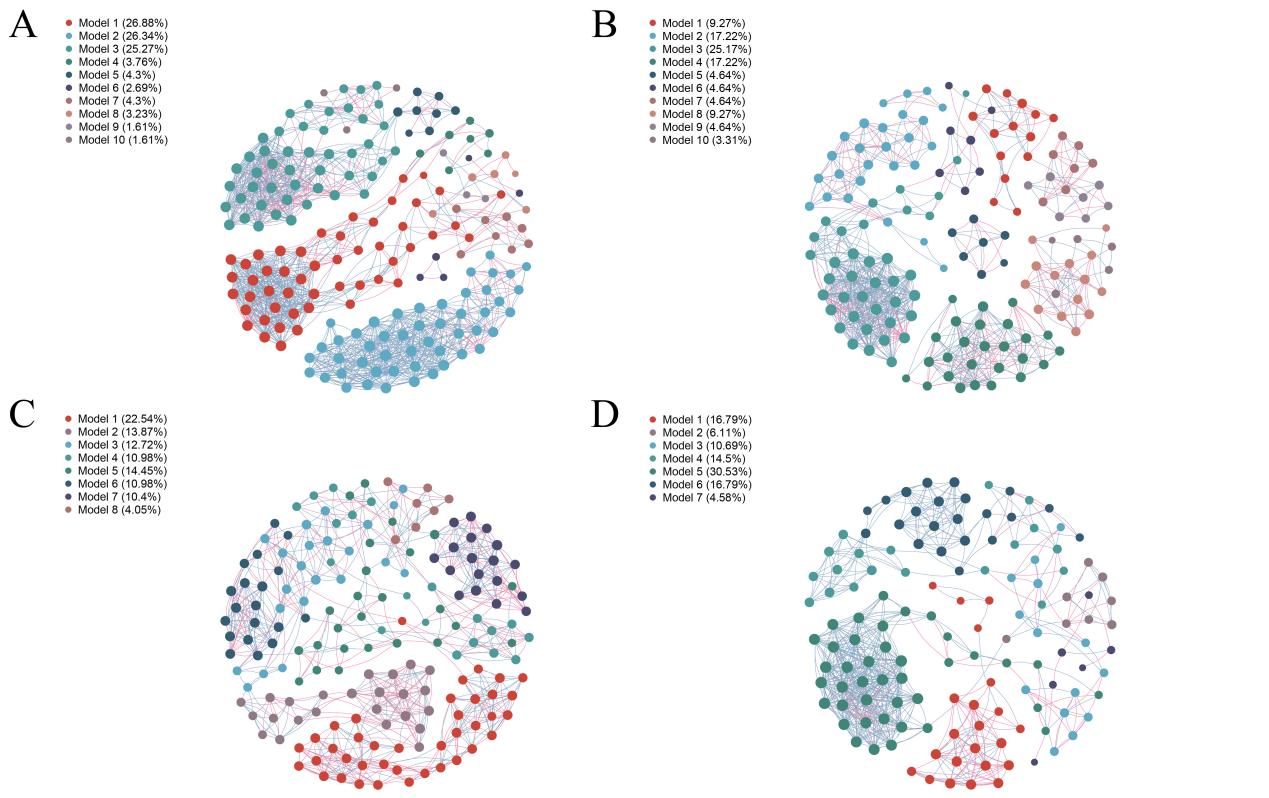


Figure S5 Characteristics of co-occurrence networks with endophytic bacterial genus. A: leaf; B: petiole; C: root; D: stem.


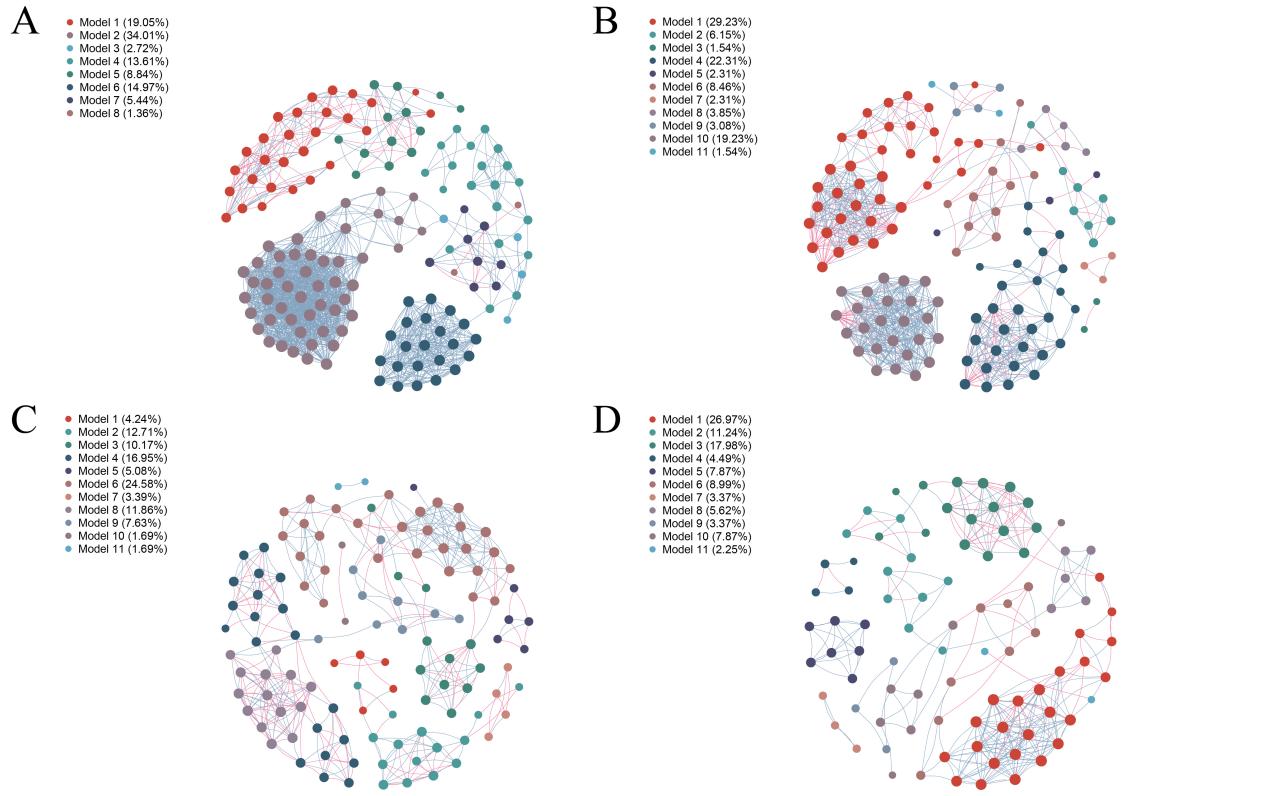


Figure S6 Characteristics of co-occurrence networks with endophytic fungal genus. A: leaf; B: petiole; C: root; D: stem.

**Data availability**

The meta-amplicon sequencing data of *A. spinulosa* are available at Figshare database. The 16S rRNA sequencing data could be accessed from <https://doi.org/10.6084/m9.figshare.25990096>, and ITS sequencing data were available in <https://doi.org/10.6084/m9.figshare.25990093>.
